# The efficacy of BLS training among fifth-year medical students—a randomized, assessor-blinded, parallel group trial

**DOI:** 10.1186/s12909-026-08606-z

**Published:** 2026-01-20

**Authors:** Gábor Fritúz, Gergely Kovács, András Kállai, Tamás Nagy, Csaba Maár, Petra Szvath, Máté Berczi, Szabolcs Fábry, Dalma Skultéti, Zsolt Iványi, János Gál, Enikő Kovács

**Affiliations:** 1 Department of Anesthesiology and Intensive Therapy, Subdepartment of Clinical Simulation, Semmelweis University, Semmelweis University, P.O.B. 2, Budapest, H-1428 Hungary; 2 Department of Anesthesiology and Intensive Therapy, Semmelweis University, P.O.B. 2, Budapest, H-1428 Hungary; 3 Department of Anesthesiology and Intensive Therapy, Institute of Anesthesiology and Perioperative Patient Care Semmelweis University, P.O.B. 2, Budapest, H-1428 Hungary; 4 Faculty of Health Sciences, Semmelweis University, P.O.B. 2, Budapest, H-1428 Hungary; 5 North Buda Szt. János Centrum Hospital, Diós árok 1-3, Budapest, 1125 Hungary; 6 Division of Military, Disaster and Law Enforcement Medicine, Semmelweis University, P.O.B. 2, Budapest, H-1428 Hungary; 7 Department of Anesthesiology and Intensive Therapy, Heart and Vascular Center, Semmelweis University, Semmelweis University, P.O.B. 2, Budapest, H-1428 Hungary

**Keywords:** Basic life support, Cardiopulmonary resuscitation, Training, Skill retention, Examination

## Abstract

**Background:**

Proper basic life support (BLS) skills are crucial for laypeople and health care professionals to increase the survival of cardiac arrest patients. A practical examination at the end of a BLS course may be beneficial for prolonging skill retention. We aimed to investigate the efficacy of our BLS training and the effect of BLS practical examinations on skill retention among fifth-year medical students compared with the effect of additional practice and continuous assessment.

**Methods:**

In this randomized, assessor-blinded, parallel group study, fifth-year medical students took a practical BLS examination (“practical examination” group) or participated in an additional 30-minute practice with continuous assessment (“additional practice” group) two weeks after a 90-minute intrahospital COVID-19 BLS training. BLS skill retention was assessed two weeks, two months and one year later, and the results of the two groups were compared. Fourteen elements of BLS were evaluated during the skill retention assessments. Descriptive statistics and Mann‒Whitney and Fisher’s exact tests were used for statistical analysis.

**Results:**

Thirty-two voluntary students were included (practical examination: *n* = 17, additional practice: *n* = 15), with no significant differences in basic characteristics (age: *p* = 0.891; gender: *p* = 0.999; previous BLS education: *p* = 0.469; previous participation in BLS: *p* = 0.678; planning to work in emergency medicine or critical care: *p* = 0.471). BLS skills were satisfactory during all skill retention assessments, except for the application of protective equipment and depth of chest compressions. More students placed surgical masks on patients’ faces in the additional practice group during the first skill retention assessment (*p* = 0.005). However, this difference disappeared over time, and both groups performed poorly in the application of protective equipment. The activation of the chain of survival and high-quality chest compressions were acceptable during all the skill retention assessments. There was no significant difference in overall BLS skill retention between the two groups (total score after two weeks: *p* = 0.764; after two months: *p* = 0.542; after one year: *p* = 0.791).

**Conclusions:**

The BLS course provided by our department was effective; however, the BLS practical examination did not offer a significant advantage in terms of skill retention compared to additional practice and continuous assessment in our student population.

**Clinical trial number:**

Not applicable

**Supplementary Information:**

The online version contains supplementary material available at 10.1186/s12909-026-08606-z.

## Background

High-quality basic life support (BLS) skills are crucial for health care workers and laypeople alike. The chain of survival summarizes the most important steps influencing the outcome of cardiac arrest and cardiopulmonary resuscitation (CPR) [1]. The first three links—1. activating the chain of survival with a call for help; 2. proper CPR with high-quality chest compressions; and 3. application of an automated external defibrillator—are all included in the BLS process, which has a significant impact on patient survival [1].

The Utstein formula for survival highlights the importance of proper education in resuscitation science to increase the quality of patient management [2]. The current guidelines of the European Resuscitation Council (ERC) include a separate chapter of recommendations on education for resuscitation [3]. It contains statements on the most important findings regarding methods used to increase the efficiency of resuscitation education. One of the problems in BLS teaching is that the learned skills deteriorate three to twelve months after the initial education if not used regularly [4–6]. To reduce the rate of skill deterioration, the guidelines recommend frequent retraining [3]. Current evidence shows that retraining leads to better BLS skills and confidence in patient management [4]. However, frequent retraining requires time, human, technical and financial resources. It remains unknown whether other methods could be effective in prolonging skill retention.

A practical or theoretical examination is frequently used to determine whether BLS course participants have achieved satisfactory proficiency. In addition, BLS practical examinations at the end of BLS training among medical students improved short-term BLS skill retention; however, this beneficial effect was not clear after six months [7, 8]. There is only limited evidence to show the effect of practical examinations on skill retention. Moreover, data comparing the effects of practical examinations and continuous assessment methods on skill retention are lacking.

Our department at Semmelweis University provides the Intensive Therapy and Anesthesiology course in a newly introduced block schedule for fifth-year medical students. One of the course goals is to train medical students in resuscitation skills with a high rate of proper skill retention. Our BLS training includes several elements designed to prolong skill retention. One of them is the application of the BLS practical examination. In addition to the usual BLS steps, we trained students on how to address some special circumstances, such as the coronavirus disease (COVID-19), during the SARS-CoV-2 outbreak.

The aim of the current study was to investigate the efficacy of our BLS training in a block schedule and to examine the efficiency in securing proper short- and long-term skill retention via BLS practical examinations compared to additional training with continuous assessment.

## Methods

### Study design and participants

This was a randomized, parallel group, single-blinded educational study investigating the effect of BLS practical examinations on skill retention compared with the effect of additional training with continuous assessment. Furthermore, the efficacy of our BLS training was also investigated. The study design and interpretation followed the CONSORT statement (Supplementary material 1). The participants were fifth-year medical students studying at Semmelweis University in the 2021/2022 academic year who participated in the Intensive Therapy and Anesthesiology three-week block organized by the Department of Anesthesiology and Intensive Therapy. Only students who applied voluntarily before the start of the course and provided written informed consent were included in the study. In addition, they were required to fill out a brief online questionnaire about their basic characteristics (gender, age, prior participation in the BLS course, and plans about future specialisation – Supplementary material 2) and to participate in at least one skill retention assessment organized at different time points after the course. There were no other exclusion criteria. The questionnaire, designed to systematically collect data on basic characteristics, underwent an internal validation process by the authors EK, AK and SzF.

The Semmelweis University Regional and Institutional Committee of Science and Research Ethics approved our study (approval number: 185/2021).

### Randomization

The students were randomized into two groups with the help of Research Randomizer Version 4.0 software (Urbaniak, G. C., & Plous, S.). The randomization of the group assignment and the enrolment of students were completed by authors EK, AK, SzF and MB. Each student received a study description and a call for participation via email one week before the first day of the Intensive Therapy and Anesthesiology block. Moreover, the students were informed and called again personally during the course orientation session directly before the first practice session of the course.

The sample size was calculated on the basis of the study results of Kromann et al., which showed that 18 students in each group would be sufficient, assuming a power of 80% and a 95% confidence interval [8].

**Fig. 1:**
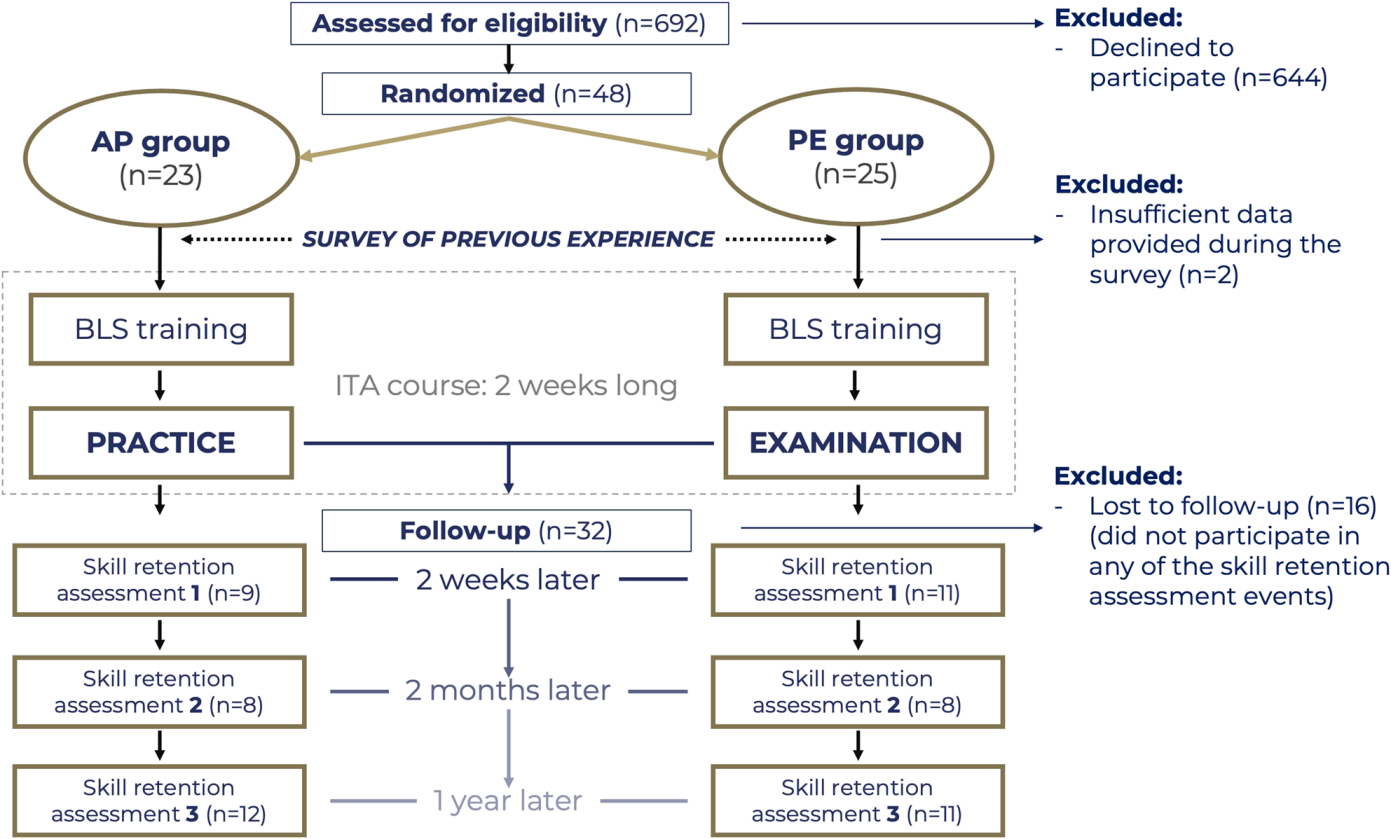
Randomization of students and the study’s flowchart. AP: Additional practice; ITA: Intensive Therapy and Anesthesiology; n: number of students; PE: Practical examination

Students randomized to the “additional practice” group took part in compulsory 90-minute BLS training sessions on the first day of the block and had an additional shorter practice session two weeks after the first training with continuous assessment evaluation by one of our instructors (authors DS, TN and CsM). Students randomized to the “practical examination” group participated in the same BLS training at the beginning of the block and took a practical BLS examination two weeks after the BLS training with no additional practice. Skill retention assessments were performed two weeks, two months and one year later by our instructors to evaluate short- and long-term BLS skill retention (authors GK, EK and PSz). In the single-blinded randomization, the students were aware of which group they belonged to; however, the instructors during BLS training and the instructors performing the skill retention assessments were blinded to who belonged to which group. Figure 1 shows the flowchart of the study.

### BLS training

Study participants with the other students in the Intensive Therapy and Anesthesiology course received a 90-minute long in-hospital BLS practice on the first day of the Intensive Therapy and Anesthesiology block according to actual ERC guidelines, supplemented with protective measures due to coronavirus disease (COVID-19) [9]. These measures included the application of simple protective equipment for self-protection (FFP2 masks and gloves), placing a surgical mask on the patient’s face before the initiation of chest compressions, checking signs of life from a specific distance and the two-person technique applied during bag‒mask ventilation.

The instructors teaching CPR at our department hold an ERC certificate or are trained following ERC educational guidelines and principles [3].

Peyton’s four-step approach is the teaching method used during BLS training, which involves the following four important steps: (1) real-time instructor demonstration of the BLS on a manikin without any explanations; (2) repeated instructor demonstration with detailed explanations followed by students’ questions, if any; (3) instructor demonstration based on students’ instructions; and (4) student demonstration with instructor help, if needed [10].

After the four-step demonstration, the students worked in pairs to practise BLS. They had to solve simple scenarios and had enough time to practise the entire algorithm. All the students received the same, unified BLS education. The key messages during training were proper initiation of the chain of survival with a call for help and high-quality chest compressions during BLS.

The teacher-to-student ratio during BLS practices was 1:6, and in some exceptional cases, it was no more than 1:8. Ambu Man C Torso™ (Ambu A/S, Copenhagen, Denmark) manikins were used during the training, examination, and skill retention assessment.

### Practical examination or additional practice with continuous assessment–intervention

Students randomized to the practical examination group took a practical BLS skills examination two weeks after the training, at the end of the practical part of the Intensive Therapy and Anesthesiology block. Students received a simple 2-minute long scenario during the examination and had to solve it in the same way as during the practice. They had no organized opportunity to practise between the BLS training and the examination. Students who failed the first time had an opportunity to retake the test on the same day after receiving brief feedback on their failure. The students not included in the study also took a practical BLS examination at the end of the block; this was the process of our usual curriculum.

Students randomized to the additional practice group participated in an additional 30-minute practice two weeks after the BLS training, at the same time as the BLS practical examination. An instructor was present during this practical session; however, he or she corrected only major mistakes (mistakes influencing calling for help or high-quality chest compressions) and evaluated the students with a continuous assessment method. It was not forbidden for the students to help and correct one another.

### Outcome

The primary outcome was defined as short-term skill retention assessed two weeks after the intervention (practical examination or additional practice). The secondary outcome was defined as long-term skill retention measured two months and one year after the interventions.

### Skill retention assessment–outcome measurement

We performed a skill retention assessment two weeks, two months and one year after the last educational intervention (practical examination or additional practice with continuous assessment) to evaluate the short- and long-term efficiency of the BLS training.

Students were invited to take part in these skill retention assessment sessions. They received two to three email notifications about the skill retention assessment, although participation was voluntary. They had to solve the same scenarios as during the practices and the practical examination. For example, “the patient suddenly collapses in front of you while walking to the hospital cafeteria”. The students had to recognize cardiac arrest, call for help and initiate CPR on the manikins. The length of a scenario was two minutes long, and the following fourteen BLS steps were evaluated by an independent instructor who did not teach the students during their BLS practice and was not involved in the practical examination or continuous assessment: (1) controlling the safety of the environment, (2) shouting for help, (3) applying self-protective equipment (gloves and FFP2 mask), (4) putting a surgical mask on the patient’s face, (5) examining consciousness, (6) checking for signs of life, (7) calling for an advanced life support team, (8) position of hands on the chest, (9) rate of chest compressions (100–120/min), (10) depth of chest compressions (5–6 cm), 11. chest release, 12. rhythm of chest compressions, 13. quality of bag–mask ventilation, and 14. maintaining a 30:2 compression-to-ventilation ratio.

Instructors used a checklist (Supplementary material 3) to indicate whether a step was implemented correctly or not and marked the exact rate and depth of chest compressions, measured with the software Ambu Man C Torso™ (Ambu A/S, Copenhagen, Denmark). A step was considered correct if it was performed according to the ERC guidelines in at least 75% during the assessment. Each of the fourteen steps was evaluated separately. If a step was correct, the student received one point (the incorrect step was evaluated with zero), and a summary score was also calculated by summing the individual BLS scores. Data regarding new experiences in BLS after our department’s BLS training were also collected (BLS training between our training and skill retention assessment, performing or witnessing real BLS between our training and skill retention assessment).

### Statistical analysis

First, the student characteristics and skill retention assessment results were compared between the additional practice and practical examination groups. In the second step, the overall results of the skill retention assessments were evaluated and compared to assess the efficacy of our BLS training.

Categorical variables are presented as numbers and percentages, whereas continuous variables are presented as median values with their corresponding interquartile ranges. Fewer than 5% of the data were missing.

The chi-square test or Fisher’s exact test (in the case of sample sizes less than five) was applied for categorical variables, and the Mann‒Whitney U test (for the comparison of additional practice and practical examination groups) or the Kruskal‒Wallis test (for the comparison of the three skill retention assessment results) was used for continuous variables. The relative risk (or risk difference if the relative risk was equal to infinity) with a 95% confidence interval was determined for categorical variables in comparing the additional practice and practical examination groups. To determine the effect size for continuous variables, Cohen’s d was calculated.

The level of significance was set at *p* < 0.05. However, owing to logistical constraints, we had to work with a smaller sample size. We also noted whether there was a tendency toward a difference between the compared groups in cases where *p* < 0.2.

Data management, statistical analysis and the creation of figures were performed using GraphPad Prism version 10.0.2 (GraphPad Software, La Jolla, CA, USA).

## Results

In the 2021/22 academic year, a total of 692 students took part in our courses. Of these, thirty-two students signed informed consent forms, were enrolled in our study and completed at least one skill retention assessment (Fig. 1). Table 1 shows the characteristics of the students included in the study. Most students took part in some type of BLS training before our course; however, fewer than half of them did so in the past year. Fewer students had real experience witnessing or performing BLS. The age, gender distribution, previous experience in BLS, previous experience in BLS education, and plans regarding the chosen speciality after university did not differ between the additional practice and practical examination groups.

**Table 1:** Basic characteristics of the fifth-year medical students included in our study

	Total	AP group	PE group	*p*	Cohen’s d or RR (95% CI)
	*n* = 32	*n* = 15	*n* = 17		
	median (IQR) or % (n)	median (IQR) or % (n)	median (IQR) or % (n)		
Age (year)	24 (23, 25)	24 (23.75, 25.5)	24 (23, 25.5)	0.891	0.167
Gender (female)	66% (21)	67% (10)	65% (11)	> 0.999	0.97 (0.59–1.60)
BLS training before our course	97% (31)	93% (14)	100% (17)	0.469	1.07 (0.94–1.23)
BLS training in the past one year before our course	44% (14)	47% (7)	41% (7)	> 0.999	0.88 (0.40–1.93)
Witnessed real BLS before our course	38% (12)	47% (7)	29% (5)	0.467	0.63 (0.25–1.57)
Took active part in real BLS before our course	22% (7)	27% (4)	18% (3)	0.678	0.66 (0.18–2.49)
Planned specialty in acute care after university	34% (11)	27% (4)	41% (7)	0.472	1.54 (0.56–4.25)

The mean values and interquartile ranges or percentages are given. The Mann‒Whitney U test or chi-square test was used

*AP *Additional practice, *BLS* Basic life support, *CI* Confidence interval, *IQR* Interquartile range, *n* Number of students, *PE* Practical examination, *RR* Relative risk

### Skill retention assessment 1

Twenty students joined the first skill retention assessment two weeks after the practical examination or additional practice. Their skill retention was acceptable for the most important steps of BLS: recognizing cardiac arrest, calling for help and the quality of chest compressions, except depth of chest compressions (Table 2). The latter were deeper than they were shallow (median value: 6.05 cm, minimum value: 4.1 cm, 25% percentile: 5.33 cm, 75% percentile: 6.53 cm, maximum value: 7.6 cm). Table 2 shows, there was no significant difference in the degree of skill retention of the most important BLS steps influencing outcomes between the additional practice and practical examination groups, except for the use of protective equipment, which was influenced by COVID-19 restrictions. Significantly more students placed a surgical mask on patients’ faces at the beginning of the BLS process in the additional practice group than in practical examination group. Moreover, there was a greater tendency to use protective equipment (gloves and masks) among the students in the additional practice group. However, the practical examination group tended to perform better in examining patient’s consciousness, the rate of chest compressions and bag–mask ventilation.

**Table 2:** Results of skill retention assessment 1, which was performed two weeks after the additional practice and continuous assessment or practical examination. The percentages of correctly performed steps are given

	Total	AP group	PE group	*p*	RR (95% CI)
	*n* = 20	*n* = 9	*n* = 11		
	% (*n*)	% (*n*)	% (*n*)		
Safety control	60% (12)	56% (5)	63% (7)	> 0.999	0.83 (0.33–2.28)
Shouting for help	90% (18)	89% (8)	91% (10)	> 0.999	0.89 (0.34–4.93)
Application of protective equipment (gloves, FFP mask)	*55% (11)*	*78% (7)*	*36% (4)*	*0.092*	*2.86 (0.95–10.6)*
Putting on a surgical mask on patients face	**65% (13)**	**100% (9)**	**36% (4)**	**0.005**	*0.69 (0.16–0.90)
Examining consciousness	*90% (18)*	*78% (7)*	*100% (11)*	*0.19*	*0.39 (0.20–1.25)*
Checking signs of life	95% (19)	89% (8)	100% (11)	0.45	0.42 (0.23–2.15)
Call for ALS team	95% (19)	100% (9)	91% (10)	> 0.999	*0.47 (-0.50-0.73)
Position of hands on the chest	95% (19)	89% (8)	100% (11)	0.45	0.42 (0.23–2.15)
Rate of chest compressions	*85% (17)*	*67% (6)*	*100% (11)*	*0.074*	*0.35 (0.17–0.93)*
Depth of chest compressions	55% (11)	63% (5)	55% (6)	> 0.999	1.02 (0.39–2.80)
Chest release	85% (17)	89% (8)	82% (9)	> 0.999	1.41 (0.45-8.00)
Rhythm of chest compressions	95% (19)	100% (9)	91% (10)	> 0.999	*0.47 (-0.50-0.73)
Quality of bag–mask ventilation	*85% (17)*	*67% (6)*	*100% (11)*	*0.074*	*0.35 (0.17–0.93)*
30:2 ratio	95% (19)	89% (8)	100% (11)	0.45	0.42 (0.23–2.15)
BLS training after our course	20% (4)	22% (2)	18% (2)	> 0.999	NA
Witnessing real BLS after our course	10% (2)	0	18% (2)	0.479	
Performing real BLS after our course	5% (1)	0	9% (1)	> 0.999	

The chi-square test or Fisher’s exact test was used

*ALS* Advanced life support, *AP* Additional practice, *BLS* Basic life support, *CI* Confidence interval, *n *Number of students, *PE* Practical examination, *RR* Relative risk

Bold: significant difference between the additional practice and practical examination groups. Italics: differential tendency between additional practice and practical examination groups

*Risk difference was calculated instead of relative risk, as relative risk would equal infinity. NA: nonapplicable, as these data are characteristics of the students and not part of the assessment

**Fig. 2:**
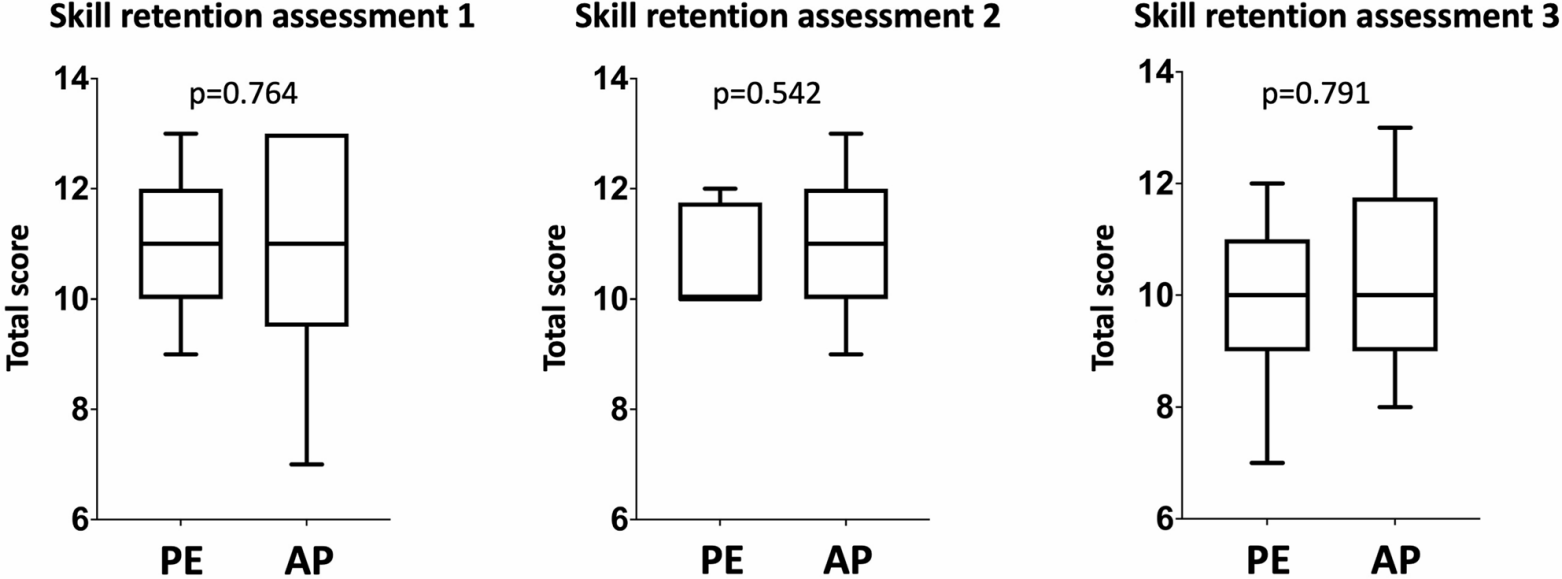
Comparison of total scores between the practical examination group and the additional practice group. There was no significant difference in the degree of skill retention between the groups. The total score was calculated as the sum of the individual BLS scores. Skill retention assessment 1 was performed two weeks, skill retention assessment 2 two months, and skill retention assessment 3 one year after the practical examination or additional training. The Mann‒Whitney test was applied. The level of significance was set at *p* < 0.05, and the level of marked difference in tendency was set at *p* < 0.2. AP: Additional practice group; PE: Practical examination group; Box-and-whiskers plot according to the Tukey method

Figure 2. shows that the total score during the first skill retention assessment was similar in both groups.

### Skill retention assessment 2

Sixteen students joined the second skill retention assessment two months after the practical examination or additional practice. Their skill retention was acceptable in almost all steps, except for their ability to control safety and apply protective equipment due to COVID-19 restrictions (Table 3). The chest compressions were also performed correctly in most cases (median value: 5.65 cm, minimum value: 4.7 cm, 25% percentile: 5.23 cm, 75% percentile: 6.00 cm, maximum value: 7.1 cm). There was no significant difference or difference in tendency between the two groups in terms of skill retention in the individual steps or in the total score (Table 3; Fig. 2).

**Table 3:** Results of skill retention assessment 2, which was performed two months after the additional practice and continuous assessment or practical examination. The percentages of correctly performed steps are given

	Total	AP group	PE group	*p*	RR (95% CI)
	*n* = 16	*n* = 8	*n* = 8		
	% (*n*)	% (*n*)	% (*n*)		
Safety control	44% (7)	50% (4)	38% (3)	> 0.999	1.29 (0.47–3.46)
Shouting for help	100% (16)	100% (8)	100% (8)	> 0.999	no difference
Application of protective equipment (gloves, FFP mask)	38% (6)	38% (3)	38% (3)	> 0.999	1.00 (0.33–2.60)
Putting on a surgical mask on patients face	69% (11)	88% (7)	50% (4)	0.282	3.18 (0.84–18.17)
Examining consciousness	88% (14)	88% (7)	88% (7)	> 0.999	1.00 (0.38–5.55)
Checking signs of life	100% (16)	100% (8)	100% (8)	> 0.999	no difference
Call for ALS team	100% (16)	100% (8)	100% (8)	> 0.999	no difference
Position of hands on the chest	88% (14)	75% (6)	100% (8)	0.467	0.43 (0.21–1.39)
Rate of chest compressions	94% (15)	100% (8)	88% (7)	> 0.999	*0.53 (-0.45-0.80)
Depth of chest compressions	81% (13)	88% (7)	75% (6)	> 0.999	1.62 (0.51–9.15)
Chest release	100% (16)	100% (8)	100% (8)	> 0.999	no difference
Rhythm of chest compressions	100% (16)	100% (8)	100% (8)	> 0.999	no difference
Quality of bag–mask ventilation	94% (15)	88% (7)	100% (8)	> 0.999	0.47 (0.25–2.39)
30:2 ratio	94% (15)	100% (8)	88% (7)	> 0.999	*0.53 (-0.45-0.80)
BLS training after our course	13% (2)	12% (1)	12% (1)	> 0.999	NA
Witnessing real BLS after our course	19% (3)	12% (1)	24% (2)	> 0.999	
Performing real BLS after our course	6% (1)	0	12% (1)	> 0.999	

The chi-square test or fisher’s exact test was used

*ALS* Advanced life support, *AP* Additional practice, *BLS* Basic life support, *CI* Confidence interval, *n* Number of students, *PE* Practical examination, *RR* Relative risk

*Risk difference was calculated instead of relative risk, as relative risk would equal infinity. NA: nonapplicable, as these data are characteristics of the students and not part of the assessment

#### Skill retention assessment 3

Twenty-three students joined the third skill retention assessment one year after the practical examination or additional training. Overall, their skill retention was acceptable in almost all steps, except for the control of safety and application of protective equipment due to COVID-19 restrictions (Table 4). In addition, only 17% of all the students performed chest compressions to an acceptable depth that was too deep (median value: 7.05 cm; minimum value: 4.4 cm; 25th percentile: 6.1 cm; 75th percentile: 7.4 cm; maximum value: 7.4 cm). There was no significant difference between the two groups in terms of skill retention in the individual steps or in the total score (Table 4; Fig. 2). There was a tendency towards a better rate of chest compressions in the practical examination group.

**Table 4:** Results of skill retention assessment 3, which was performed one year after the additional practice and continuous assessment or practical examination. The percentages of correctly performed steps are given

	Total	AP group	PE group	*p*	RR (95% CI)
	*n* = 23	*n* = 12	*n* = 11		
	% (*n*)	% (*n*)	% (*n*)		
Safety control	22% (5)	25% (3)	18% (2)	> 0.999	1.20 (0.43–2.48)
Shouting for help	96% (22)	100% (12)	91% (10)	0.478	*0.55 (-0.42-0.70)
Application of protective equipment (gloves, FFP mask)	44% (10)	50% (6)	36% (4)	0.68	1.30 (0.58–2.89)
Putting on a surgical mask on patients face	35% (8)	33% (4)	36% (4)	> 0.999	0.94 (0.37–2.03)
Examining consciousness	96% (22)	92% (11)	100% (11)	> 0.999	0.50 (0.31–2.50)
Checking signs of life	100% (23)	100% (12)	100% (11)	> 0.999	no difference
Call for ALS team	96% (22)	92% (11)	100% (11)	> 0.999	0.50 (0.31–2.50)
Position of hands on the chest	87% (20)	83% (10)	91% (10)	> 0.999	0.75 (0.38–2.55)
Rate of chest compressions	*65% (15)*	*50% (6)*	*82% (9)*	*0.193*	*0.53 (0.25–1.17)*
Depth of chest compressions	17% (4)	25% (3)	9% (1)	0.59	1.58 (0.58–3.05)
Chest release	96% (22)	100% (12)	91% (10)	0.478	*0.55 (-0.42-0.78)
Rhythm of chest compressions	100% (23)	100% (12)	100% (11)	> 0.999	no difference
Quality of bag–mask ventilation	96% (22)	100% (12)	91% (10)	0.478	*0.55 (-0.42-0.78)
30:2 ratio	78% (18)	83% (10)	73% (8)	0.64	1.39 (0.58–4.96)
BLS training after our course	9% (2)	8% (1)	9% (1)	> 0.999	NA
Witnessing real BLS after our course	30% (7)	42% (5)	18% (2)	0.371	
Performing real BLS after our course	30% (7)	33% (4)	27% (3)	> 0.999	

The chi-square test or Fisher’s exact test was used

*ALS* Advanced life support, *AP* Additional practice, *BLS* Basic life support, *CI* Confidence interval, *n* Number of students, *PE* Practical examination, *RR* Relative risk

*Risk difference was calculated instead of relative risk, as relative risk would equal infinity. NA: nonapplicable, as these data are characteristics of the students and not part of the assessment

### Changes in skill retention

Table 5 presents a comparison of all the students’ results during the three skill retention assessments. Students had satisfactory and consistent performance in nine of the fourteen examined steps (shouting for help, examining consciousness, checking for signs of life, calling for the ALS team, position of hands during chest compression, chest release, rhythm of chest compression, quality of bag–mask ventilation and maintaining a 30:2 ratio). At the beginning of the BLS assessment, only 60% of participants accomplished the safety control in the first skill retention assessment, and this performance declined significantly over time. Protective equipment was applied poorly during all the skill retention assessments. The placement of a surgical mask on the patient’s face was absent in approximately 30% of the patients during the first and second skill retention assessments and in 65% of the patients during the third skill retention assessment. Maintaining a proper rate of chest compressions was poorer one year after the training; however, the amount of deviation from the correct rate was not significant. The depth of chest compressions was also poor during the first assessment and even poorer during the third skill retention assessment; however, compressions were deeper than the expected depth, which was not shallow.

The total scores were similar across all three skill retention assessments (SRA 1 median: 11, interquartile ranges: 10, 12; SRA2 median: 10.5, interquartile ranges: 10, 12; SRA 3 median: 10, interquartile ranges: 9, 11; *p* = 0.225; Cohen’s d = 0.268).

**Table 5:** Comparison of the skill retention assessment results. The percentages of correctly performed steps and the values of the depth and rate of chest compression are given

	SRA 1	SRA 2	SRA 3	*p*
	*n* = 20	*n* = 16	*n* = 23	
	% (n) or median (IQR)	% (n) or median (IQR)	% (n) or median (IQR)	
Safety control	**60% (12)**	**44% (7)**	**22% (5)**	**0.037**
Shouting for help	90% (18)	100% (16)	96% (22)	0.390
Application of protective equipment (gloves, FFP mask)	55% (11)	38% (6)	44% (10)	0.555
Putting on a surgical mask on patients face	*65% (13)*	*69% (11)*	*35% (8)*	*0.055*
Examining consciousness	90% (18)	88% (14)	96% (22)	0.638
Checking signs of life	95% (19)	100% (16)	100% (23)	0.371
Call for ALS team	95% (19)	100% (16)	96% (22)	0.676
Position of hands on the chest	95% (19)	88% (14)	87% (20)	0.641
Rate of chest compressions	*85% (17)*	*94% (15)*	*65% (15)*	*0.072*
Rate of chest compressions (rate/minute)	*108 (103*,* 116)*	*112 (109*,* 118)*	*103 (98*,* 116)*	*0.081*
Depth of chest compressions	**55% (11)**	**81% (13)**	**17% (4)**	**0.0003**
Depth of chest compressions (cm)	**6.05 (5.33**,** 6.53)**	**5.65 (5.23**,** 6.00)**	**7.05 (6.10**,** 7.40)**	**0.004**
Chest release	85% (17)	100% (16)	96% (22)	0.172
Rhythm of chest compressions	95% (19)	100% (16)	100% (23)	0.371
Quality of bag–mask ventilation	85% (17)	94% (15)	96% (22)	0.426
30:2 ratio	95% (19)	94% (15)	78% (18)	0.171

The chi-square test or Kruskal‒Wallis test was used

*ALS* Advanced life support; n: number of students, *SRA 1* Skill retention assessment two weeks after the additional training or practical examination, *SRA 2* Skill retention assessment two months after the additional training or practical examination, *SRA 3* Skill retention assessment one year after the additional training or practical examination.

Bold: statistically significant. Italics: indicates a differential tendency between the skill retention assessment results

## Discussion

Improving BLS skill retention among laypeople and health care professionals is one of the most important tasks for improving cardiopulmonary resuscitation efficiency and increasing survival rates after cardiac arrest. We investigated the effect of practical examinations on skill retention compared with additional training with continuous assessment. Furthermore, the efficacy of our 90-minute BLS training held for health care professionals (fifth-year medical students) containing some extra elements targeting the prolongation of skill retention was also examined. Our results suggest that the BLS practical examination did not confer a significant advantage over additional practice with continuous assessment in this sample. In addition, we found that our BLS training was effective, as both short- and long-term skill retention were acceptable in the most important BLS steps: activating the chain of survival and performing high-quality chest compressions. However, some skills related to safety during patient management (the control of a safe environment and the application of protective equipment) were poor overall and deteriorated over time. Moreover, students in the additional practice group had better short-term retention of these skills, yet this difference disappeared with time. The uniqueness of the current study is the comparison of the effects of BLS practical examinations or additional BLS practices with continuous assessments of skill retention. Moreover, we investigated this effect in the newly developed BLS training program held during a three-week block schedule for medical students. During the era following the COVID-19 pandemic, the COVID-related modifications to BLS practices also provide important lessons.

It is already well known that BLS skills deteriorate within three to twelve months if BLS is not practised regularly [4–6]. Moreover, these skills should be updated routinely as the guidelines may change. There are several methods aimed at prolonging skill retention. The actual ERC guidelines on education recommend learner-adapted programs, technology-enhanced learning and annual short competency refreshers [3]. Refresher courses are among the key tools in BLS skill maintenance, and lifelong learning is promoted. Additionally, a randomized trial showed that taking a practical examination at the end of a BLS course can be more effective in maintaining short-term BLS skills (skill retention after two weeks was examined) than additional 30-minute practices at the end of a course among medical students [8]. However, this advantage was not evident after six months [7]. In contrast, we found no major difference in the long- or short-term performance between the practical examination group and the additional practice group. However, we must outline some differences between the previous studies and the current trial. The former studies evaluated only a global picture of BLS skills and did not evaluate BLS steps separately. In contrast, we measured the appropriateness of 14 important steps during BLS. Moreover, we compared practical examinations held two weeks after the additional practice was enhanced with continuous assessment. In the studies of Kromann et al., practical examinations were organized directly at the end of the practice.

A randomized noninferiority trial showed that repetitive sessions of formative self-testing may be a potential method to refresh skills [11]. We need to emphasize that testing is noninferior to additional practices in this study.

Additionally, our research group previously examined the effects of BLS practical examinations and the timing of BLS examinations on skill retention among medical students in an observational study. The results showed that practical examinations at the end of BLS training did not influence skill retention measured two months after the examination; however, if students took the practical examinations three months after the training, during the examination period, skill retention improved [12]. However, the university examination period ended after the introduction of the new block schedule in the 2020/21 academic year at Semmelweis University. A new curriculum for the Intensive Therapy and Anesthesiology course was created, containing a two-week practical part and a one-week examination part. We decided to start the block with BLS training followed by a BLS practical examination two weeks later to provide some time between the practice and the examination on the basis of the results of our previous study. However, an important question was whether an additional practice supplemented by continuous assessments of student performance could be as effective as a practical examination in prolonging skill retention. According to our results, there were no major differences in the steps influencing survival between the additional practice and practical examination groups.

There is clear evidence that activating the chain of survival with early calls for help and high-quality chest compressions can increase the survival rate after cardiac arrest [1, 13]. High-quality chest compressions can be characterized as follows: the hand is positioned in the middle of the chest, the rate of chest compressions is 100–120/min, the depth of chest compressions is 5–6 cm, the rhythm of chest compressions is constant, and the release of the chest is secured [14]. If the depth of chest compression is too shallow, the efficacy of the chest compression is affected [15, 16]. Deeper chest compressions are efficient; however, they may increase the incidence of injuries [17]. The importance of early calls for help and high-quality chest compressions were highlighted during BLS practices, and students failed the BLS practical examination if these steps were performed poorly.

Both short- and long-term skill retention were satisfactory in terms of activating the chain of survival and the quality of chest compressions after completing our BLS training, except for chest compressions, which were somewhat too deep.

The most important strengths of our BLS training are the optimal instructor-to-student ratio, the application of Peyton’s four-step method and the unified theme of the practice. It was already shown that the maximum group size enabling the correction of more than 80% of errors during training is six persons [18]. Peyton’s four-step approach was also shown to be effective when used in small groups [19].

We must highlight that almost all the students participated in some type of BLS training before Intensive Therapy and Anesthesiology course, meaning our BLS training was already a repeated session. The major novelties introduced during BLS training were protective measures as a part of COVID-19 BLS. COVID-19 BLS skills were taught because the COVID-19 pandemic in Hungary was still present during the 2021/22 academic year. These skills were clearly poorer during the skill retention assessments. We believe that these skills receive less attention during the practices and practical examinations; however, the safety of health care professionals is also important, and we are planning to strengthen the education of these skills in the future. The lessons learned from the COVID-19 era can also be used in the future to strengthen other aspects of our practice, such as highlighting safety measures during patient care.

We could not demonstrate an advantage of the BLS practical examination taken two weeks after the training over additional practice with continuous assessment. The potential benefit of practical examinations is based on the so-called “testing effect”, according to which the repeated retrieval of memories during an examination improves skill retention better than repeated study does [20, 21]. Importantly, in the current study, the students in the additional practice group received feedback on their performance in the framework of a continuous assessment method. To clarify the proper circumstances under which the practical examination or additional practice and continuous assessment would be more beneficial requires further investigation.

### Strengths and limitations of the study

This was a randomized parallel group study evaluating not only overall BLS skill retention but also the most important individual BLS steps. We investigated both short- and long-term skill retention.

However, several limitations need to be considered. The most important limiting factor is the small sample size. Participation in the study and participation in the skill retention assessments were voluntary. The one third of enrolled students was excluded because they did not join any of the skill retention assessment events despite our efforts to provide flexible opportunities to take the assessment. Additionally, we could not include more students from the following academic years, as our curriculum changed, and we would not have been able to compare the results without further bias. This may have resulted in a lower statistical power and an increased risk of type II errors. To refine this bias, we marked differences even in cases where *p* < 0.2.

The voluntary nature of participation may introduce selection bias; however, our university regulations do not permit interventions to be applied differently.

We tried to secure the same instructors for the BLS training and practical examinations; however, this was not always possible. Although the BLS practice theme was unified and our instructors were trained on the basis of the ERC standards, we cannot rule out the possibility of some differences in education related to instructors’ characteristics and experience.

We collected data from students on their previous experience in BLS and BLS education before our course; however, a skill assessment before BLS training would also be useful in the future.

## Conclusions

Proper BLS skills are important for laypeople and health care workers to improve cardiac arrest survival. However, these skills deteriorate over time if not used. We investigated the efficacy of BLS training held for fifth-year medical students. We found no major advantage of the BLS practical examination two weeks after training over additional training with continuous assessment.

## Supplementary Information

Supplementary Material 1

Supplementary Material 2

Supplementary Material 3

## Data Availability

The datasets supporting the conclusions of this article can be requested from the author Enikő Kovács (kovacs.eniko2@semmelweis.hu).

## Abbreviations

ALS: Advanced life support
AP: Additional practice
BLS: Basic life support
CPR: Cardiopulmonary resuscitation
ERC: European Resuscitation Council
IQR: Interquartile range
PE: Practical examination
SRA: Skill retention assessment
