# Supplementary material for: The efficacy of BLS training among fifth-year medical students—a randomized, assessor-blinded, parallel group trial
Source: BMC Med Educ. 2026 Jan 20;26:286. doi: 10.1186/s12909-026-08606-z (PMC12910728; doi:10.1186/s12909-026-08606-z)
Supplement: Supplementary file 2 — Supplementary Material 2 [file 12909_2026_8606_MOESM2_ESM.pdf]

Questionnaire on participants' basic characteristics

Dear Student,

Thank you for taking part in our study. After signing the informed consent, as a next step, please fill out this form to collect your demographic data and some information about your previous basic life support education and experience. All of your data will be managed confidentially. To fill out the questionnaire you need approx. 5 minutes.

If you have any questions, do not hesitate to contact me: kovacs.eniko2@med.semmelweis-univ.hu

Enikő Kovács

1. Provide your Neptun-code:

2. Provide your gender:
   a. male b. female c. other

3. Provide your age:

4. Have you ever received any basic life support (BLS) education?
   a. yes b. no

5. If you received BLS education prior to our course, please provide when it happened:
   a. in 1 year b. in 2-5 years c. in 5-10 years
   d. more than 10 years ago

6. Have you ever seen BLS in life before?
   a. Yes b. No

7. Have you previously participated in BLS as a provider?
   a. Yes b. No

8. If you have seen BLS in life or have participated in BLS as a provider, please provide when it happened:
   a. in 1 year b. in 2-5 years c. in 5-10 years

d. more than 10 years ago

9. If you already know what specialty you choose after graduation, mark the answer that best describes you:
    a. I would like to choose anesthesiology and intensive therapy
    b. I would like to choose emergency medicine
    c. I would like to choose cardiology
    d. I would like to work in a conservative profession not listed above (eg. internal medicine, neurology, psychiatry, etc.)
    e. I would like to work in a surgical profession (eg. general surgeon, gynecologist, orthopedist, traumatologist, etc.)
    f. I will be leaving medical profession
    g. I do not know yet what specialisation I choose
