# Supplementary material for: The efficacy of BLS training among fifth-year medical students—a randomized, assessor-blinded, parallel group trial
Source: BMC Med Educ. 2026 Jan 20;26:286. doi: 10.1186/s12909-026-08606-z (PMC12910728; doi:10.1186/s12909-026-08606-z)
Supplement: Supplementary file 3 — Supplementary Material 3 [file 12909_2026_8606_MOESM3_ESM.pdf]

The impact of practical examination on BLS skill retention - checklist

Skill retention assessment nb. 1

Date:

Student's Neptun code:

Name of the assessor:

Assessed 14 steps (Please, if the student completed the given step correctly in at least 75% during the scenario, add number 1 to the given step and if the stpe was inappropriate, add number 0):

Controlling the safety of environment
Shouting for help
Application of self-protective equipment
Putting on a surgical mask on patients' face
Examining consciousness
Checking signs of life
Call for advanced life support team
Chest compression:
Position fo hands on the chest
Rate: 100-120/min
Depth: 5-6 cm
Total chest release
Rhythm of chest compressions
Quality of bag-mask ventilaion
30:2 ratio
Comments
